# *Angelica purpurascens* (Avé-Lall.) Gilli. Essential Oil Improved Brain Function via Cholinergic Modulation and Antioxidant Effects in the Scopolamine-Induced Zebrafish (*Danio rerio*) Model

**DOI:** 10.3390/plants11081096

**Published:** 2022-04-18

**Authors:** Razvan Stefan Boiangiu, Eyup Bagci, Gabriela Dumitru, Lucian Hritcu, Elena Todirascu-Ciornea

**Affiliations:** 1 Department of Biology, Faculty of Biology, Alexandru Ioan Cuza University of Iasi, 700506 Iasi, Romania; razvan.boiangiu@uaic.ro (R.S.B.); ciornea@uaic.ro (E.T.-C.); 2 Department of Biology, Faculty of Science, Firat University, 23119 Elazig, Turkey; ebagci@firat.edu.tr

**Keywords:** *Angelica purpurascens*, scopolamine, acetylcholinesterase, oxidative damage, memory

## Abstract

*Angelica purpurascens* (Avé-Lall.) Gilli. is a medicinal plant that displays antioxidant, anticholinesterase, and neuroprotective properties. The effect of *A. purpurascens* essential oil (APO) on memory impairments and brain oxidative stress in zebrafish (*Danio rerio*) treated with scopolamine (Sco), as well as the underlying mechanism involved, were investigated in this study. Exposure to Sco (100 μM) resulted in anxiety in zebrafish, as assessed by the novel tank diving test (NTT), whereas spatial memory and novelty response dysfunctions, as evidenced by the Y-maze test and novel object recognition test (NOR), were noticed. When zebrafish were given Sco and simultaneously given APO (25 and 150 μL/L, once daily for 13 days), the deficits were averted. An increase in brain antioxidant enzymes, a reduction of lipid peroxidation, and protein oxidation were linked to this impact. Furthermore, acetylcholinesterase (AChE) activity was significantly reduced in the brains of APO-treated zebrafish. The main detected components in the APO composition were β-phellandrene (33.80%), sabinene (6.80%), α-pinene (5.30%), germacrene-D (4.50%), α-phellandrene (4.20%), and *p*-cymene (3.80%) based on gas chromatography–mass spectrometry (GC-MS) investigations. Our findings show that APO’s beneficial effect in a zebrafish model of Sco-induced memory impairment is mediated through multiple mechanisms, including the restoration of cholinergic system function and the improvement of the brain antioxidant state. As a result, APO could be employed as a potential source of bioactive molecules with useful biological properties and medicinal uses.

## 1. Introduction

Alzheimer’s disease (AD), which accounts for most dementia cases, is marked by a deterioration in cognitive function and gradual memory loss due to impairment in hippocampus neurogenesis [1]. Amyloid plaques, which are formed by deposits of amyloid β-proteins (Aβ), and neurofibrillary tangles, which are formed by the aggregation of hyperphosphorylated tau proteins, are the two principal pathological hallmarks of AD [2]. The pathogenesis of AD also includes neuroinflammation, oxidative stress, decreased synaptic plasticity, and cholinergic dysfunction [3]. Memory loss and cognitive deficits are most strongly linked to changes in cholinergic system activities [4]. Scopolamine’s (Sco) cholinergic muscarinic receptor antagonistic action, which inhibits learning and memory in animal models, makes it a helpful pharmacological tool for the study of learning and memory deficits [5]. Moreover, Sco increased acetylcholinesterase (AChE) activity and enhanced oxidative stress [6]. Tacrine (TAC), donepezil, and rivastigmine are types of AChE inhibitors that have been used to treat patients with AD [7]. All these drugs, however, have limited efficacy and harmful side effects as the disease progresses, making them ineffective against AD [8]. As a result, alternative and complementary therapy for AD must be created.

The genus *Angelica* L. belongs to the Apiaceae (Umbelliferae) family, which includes roughly 90 species of biennial perennial herbs found in Asia, Europe, and North America [9]. It has been reported that three *Angelica* species: *A. archangelica* L., *A. sylvestris* L., and *A. purpurascens* (Avé-Lall.) Gilli. grows in Turkey [10]. The Apiaceae family member is defined by a high concentration of monoterpenes, sesquiterpene hydrocarbons, and phenolic chemicals [11] and are proved to have positive effects on the central nervous system [12]. Adsersen et al. [13] reported that some species belonging to the Apiaceae (*Carum carvi* L., *Petroselinum crispum* (Mil.) Nym.ex A.W.Hill., and *Pimpinella anisum* L.) used in Danish folk medicine displayed memory-supporting properties and AChE inhibitory activity. Moreover, Karakaya et al. [10] revealed the anti-AChE and antioxidant properties of isolated compounds from *A. purpurascens* (Avé-Lall.) Gilli. fruits such as stigmasterol, β-sitosterol, bergapten, and oxypeucedanin.

There has been no evidence that *A. purpurascens* (Avé-Lall.) Gilli. protects zebrafish memory from Sco-induced memory deficits by altering cholinergic and antioxidant pathways. The phytochemical content of *A. purpurascens* (Avé-Lall.) Gilli. essential oil was investigated, and how it affected anxiety, cognitive performance, and brain antioxidant capacity in a Sco-induced zebrafish model was assessed.

## 2. Results and Discussion

### 2.1. Characterization of the Angelica Purpurascens Essential Oil

In the chemical analysis of the sample used, 12 individual components were determined, representing 66.30% of the total composition. The main constituents identified in the APO were (%): β-Phellandrene, 33.80; Sabinene, 6.80; α-Pinene, 5.30; Germacrene-D, 4.50; α-Phellandrene, 4.20; *p*-Cymene, 3.80. Table 1 presents the major chemical constituents identified in APO.

Some of the chemical compounds of our essential oil samples, identified in the aerial parts, such as α-pinene, sabinene, limonene, β-elemene, and germacrene-D, were reported by Turkucar et al. [14] that evidenced α-bisabolol (22.93%), cubebol (14.39%), α-pinene (11.63%), α-limonene (9.41%), aromadendrene-dehydro (4.64%), β-elemene (4.56%), sabinene (4.48%), and germacrene-D (4.47%) as the main components in the case of *A. purpurascens* root essential oil analysis. Karakaya et al. [10] reported two main compounds, α-pinene (33.70%) and β-phellandrene (4.3%), from the essential oil of the aerial parts of *A. purpurascens*. Moreover, Nivinskiene et al. [15] reported the presence of α-pinene (15.70–20.80%) as the dominant component in two localities, with β-phellandrene (13.80–18.50%) and α-pinene (11.40–15.0%) registered in the third locality found in the chemical composition of the essential oil of *A. archangelica* L. roots growing wild in Lithuania. According to Irshad et al. [16], the essential oil of the *A. glauca* entire plant obtained from Jammu and Kashmir principally contains α-phellandrene (18.00%), trans-carveol (16.40%), β-pinene (14.00%), β-caryophyllene (8.60%), and β-caryophyllene oxide (8.00%). According to Cavaleiro et al. [17], the most prevalent components in the essential oil of *A. major* are α-pinene (21.80%) and cis-β-ocimene (30.40%). Our essential oil has a chemical composition that is like that of other authors who believe it possesses memory-enhancing and antioxidant qualities, based on these findings.

### 2.2. Effects on Zebrafish NTT Response

After the administration of APO (25 and 150 μL/L), the zebrafish did not exhibit behavioral abnormalities or other markers of toxicity and mortality, demonstrating the safety profile of the APO dosages.

**Figure 1:**
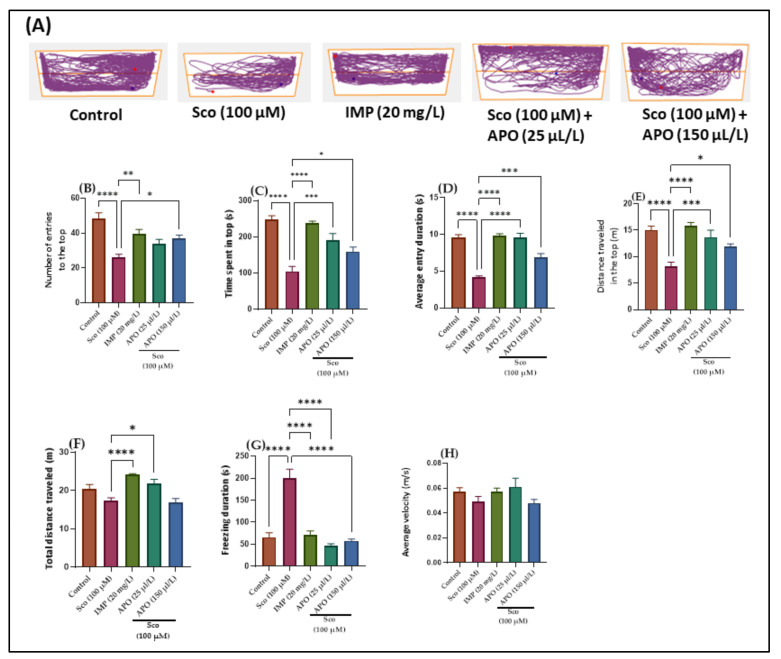
NTT results for *Angelica purpurascens* essential oil (APO: 25 and 150 μL/L). (**A**) Representative tracking locomotion patterns; (**B**) Number of entries to the top; (**C**) Time spent in top (s); (**D**) Average entry duration (s); (**E**) Distance traveled in the top (m); (**F**) Total distance traveled (m); (**G**) Freezing duration (s); (**H**) Average velocity (s). Data are expressed as means ± S.E.M. (*n* = 10). * *p* < 0.01, ** *p* < 0.001, *** *p* < 0.0001, and **** *p* < 0.00001 (Tukey’s post hoc analyses). Imipramine (IMP, 20 mg/L) was used as reference positive drug.

The effects of Sco (100 μM) and APO (25 and 150 μL/L) on anxiety-like behavior in the NTT test are depicted in Figure 1. Within the NTT, the discrepancies between the top and bottom zones in swimming traces are shown in the typical locomotion tracking patterns (Figure 1A). According to the one-way ANOVA, the treatment had a significant impact on the number of entries to the top of the tank in the NTT (F (4, 45) = 10.65 *p* < 0.0001). (Figure 1B). In addition, one-way ANOVA revealed significant overall differences in the time spent in the top (F (4, 45) = 19.06, *p* < 0.001) (Figure 1C), the average entry duration (F (4, 45) = 35.24, *p* < 0.0001) (Figure 1D), the distance traveled in the top (F (4, 45) = 12.67, *p* < 0.0001) (Figure 1E), the total distance traveled (F (4, 45) = 10.72, *p* < 0.0001) (Figure 1F), and the freezing duration (F (4, 45) = 30.43, *p* < 0.0001) (Figure 1G).

Zebrafish treated with imipramine (IMP, 20 mg/L), a tricyclic antidepressant [18] used as a positive control, exhibited high preference for the top zone of the tank (*p* < 0.001) (Figure 1B) and spent more time in the top of the tank (*p* < 0.00001) (Figure 1C,D) as compared to Sco alone-treated fish. In addition, IMP-exposed zebrafish displayed hyperlocomotion, as evidenced by the increased distance traveled in the top (*p* < 0.00001) (Figure 1E,F) and the decreased freezing duration (*p* < 0.00001) (Figure 1G) compared with the Sco-induced model. These results suggested that IMP had an anxiolytic profile. In comparison to the control group, Sco-treated zebrafish rarely approached the tope zone of the tank (*p* < 0.00001) (Figure 1B) and spent less time there (*p* < 0.00001) (Figure 1C,D). It is possible that Sco caused a lot of anxiety. Additionally, the Sco treatment had a hypolocomotor effect, lowering the distance traveled in the top (*p* < 0.00001) (Figure 1E) and extending the freezing time (*p* < 0.00001) (Figure 1G). Furthermore, APO treatment prevented the anxiogenic effect of Sco dose-dependently by increasing the number of entries in the tope zone of the tank (*p* < 0.01 for 150 μL/L) (Figure 1B), the time spent at the tope zone of the tank (*p* < 0.0001 for 25 μL/L and *p* < 0.01 for 150 μL/L) (Figure 1C), and the average entry duration (*p* < 0.00001 for 25 μL/L and *p* < 0.0001 for 150 μL/L) (Figure 1D) compared to the Sco alone-treated group. When compared to the Sco alone group, APO exposure prevented the Sco-induced hypolocomotor effect by increasing the distance traveled in the top (*p* < 0.0001 for 25 μL/L and *p* < 0.01 for 150 μL/L) (Figure 1E), the total distance traveled (*p* < 0.01 for 25 μL/L) (Figure 1F), and by decreasing the freezing duration (*p* < 0.00001 for 25 μL/L and *p* < 0.00001 for 150 μL/L) (Figure 1G). There were no noticeable effects on average velocity (Figure 1H).

Our results corroborate previous research, which found that administering *Angelica* extracts had antidepressant effects. Shen et al. [19] found that *A. sinensis* extracts have an antidepressant effect on chronic unpredictable mild stress-induced depression in rats, which is mediated by the activation of the brain-derived neurotrophic factor (BDNF) signaling pathway. Cao et al. [20] used the 5-hydroxytryptamine system to show that imperatorin from *A. dahurica* had an antidepressive-like effect in prenatally stressed offspring rats. In three murine tests of anxiety with diazepam as a positive anxiolytic control, Chen et al. [21] discovered the anxiolytic efficacy of *Angelica* essential oil. In addition, Lee et al. [22] showed that *A. gigas* reduced depression-like symptoms in rats following chronic corticosterone injection.

The main components of the essential oil employed in this investigation have been shown to have anxiolytic properties. Saeedipour and Rafieirad [21] found that α-pinene had a down-regulating effect on anxiety responses in mice which was amplified by binding to GABA_A_ receptors. According to Helwein et al. [23] and Koyama and Heinbockel [24], another chemical, sabinene, has a considerable anxiolytic effect that is mediated through the GABAergic system. Our findings suggested that APO could prevent Sco-induced anxiety behavior in zebrafish via modulating GABAergic system activity, based on these findings.

### 2.3. Effects on Zebrafish Y-Maze and NOR Response

**Figure 2:**
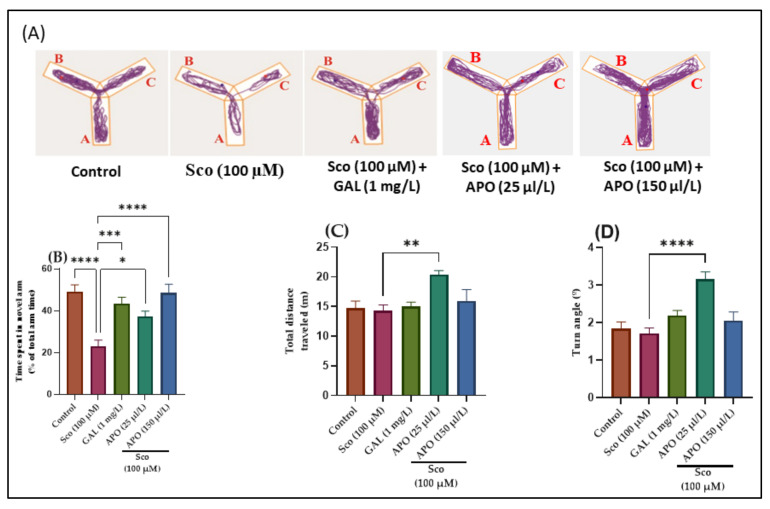
Y-maze results for *Angelica purpurascens* essential oil (APO: 25 and 150 μL/L). (**A**) Representative tracking locomotion patterns; (**B**) Time spent in the novel arm (% of total arm time); (**C**) Total distance traveled (m); (**D**) Turn angle (°). Data are presented as means ± S.E.M. (*n* = 10). * *p* < 0.01, ** *p* < 0.001, *** *p* < 0.0001, and **** *p* < 0.00001 (Tukey’s post hoc analyses). Galantamine (GAL, 1 mg/L) was used as reference positive drug.

The effects of Sco (100 μM) and APO (25 and 150 μL/L) treatments on the Y-maze novelty response and locomotion are shown in Figure 2. Changes in swimming traces in the Y-maze are depicted in Figure 2A. It was shown that Sco administration resulted in reduced exploration of the novel arm in the Y-maze test, implying an impairment on a Y-maze response to novelty. The one-way ANOVA revealed a significant overall effect of the treatment in the Y-maze on the time spent in the novel arm (% of total arm time) (F (4, 45) = 11.05, *p* < 0.0001)) (Figure 2B), the total distance traveled (F (4, 45) = 4.44, *p* < 0.001)) (Figure 2C), and the turn angle (F (4, 45) = 10.56, *p* < 0.0001)) (Figure 2D).

Administration of galantamine (GAL, 1 mg/L), a cholinesterase inhibitor [25], increased the preference for the novel arm (*p* < 0.0001) (Figure 2B) compared with the Sco-induced zebrafish model, suggesting an increase of the response to novelty. Moreover, nonsignificant improvement of locomotion following GAL treatment was noticed (Figure 2C,D) as compared to Sco group. Sco treatment reduced the time spent in the novel arm (*p* < 0.00001) (Figure 2B) when compared to the control group, indicating that the response to novelty was reduced. Moreover, Sco treatment induced a nonsignificant reduction in the locomotory activity in the Y-maze test, as evidenced by the total distance traveled (*p* > 0.05) (Figure 2C) and the turn angle (*p* > 0.05) (Figure 2D) compared to the control group.

In contrast to the Sco alone-treated zebrafish, APO administration dramatically increased the time spent in the novel arm of the tank (*p* < 0.0001 for 25 μL/L and *p* < 0.00001 for 150 μL/L) (Figure 2B), showing positive effects on the response to novelty. Furthermore, as compared to Sco alone-treated zebrafish, APO administration increased locomotion as indicated by significant increases in the total distance traveled (*p* < 0.0001 for 25 μL/L) (Figure 2C) and the turn angle (*p* < 0.00001 for 25 μL/L) (Figure 2D).

**Figure 3:**
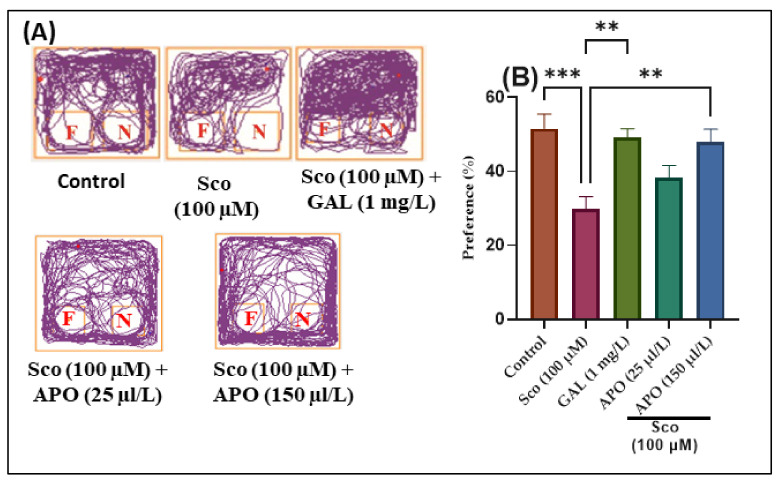
NOR results for *Angelica purpurascens* essential oil (APO: 25 and 150 μL/L) (**A**) Representative locomotion tracking patterns; (**B**) Preference (%). Data are presented as means ± S.E.M. (*n* = 10). ** *p* < 0.001 and *** *p* < 0.0001 (Tukey’s post hoc analyses). Galantamine (GAL, 1 mg/L) was used as reference positive drug.

The effects of the Sco (100 μM) and APO (25 and 150 μL/L) administration on NOR recognition memory and locomotion are illustrated in Figure 3. In the NOR test, representative locomotion tracking patterns revealed changes in swimming behavior (Figure 3A). It was discovered that the Sco treatment increased the preference for familiar objects (F) over novel objects (N), implying a problem with recognition memory.

According to one-way ANOVA, the treatment had significant overall effects on preference percentages (F (4, 45) = 7.40, *p* < 0.0001) (see Figure 3). In the NOR test, zebrafish treated with GAL displayed a high preference for the novel object (N) (*p* < 0.0001) (Figure 3B) as compared to the Sco group. In comparison to the control group, zebrafish treated with Sco had reduced preference percentages for the N (*p* < 0.0001) (Figure 3B), implying impairment of recognition memory. Furthermore, APO treatment (25 and 150 μL/L) raised preference percentages in Sco-induced zebrafish (*p* < 0.001 for 150 μL/L) (Figure 3B), implying a memory-enhancing pattern.

Our findings showed that APO had cognitive-enhancing properties. This is in accordance with previous studies, which indicated that taking *Angelica* extracts significantly reduced memory loss. Kim et al. [26] found that *A. gigas* improved spatial memory in specific tasks such as the Y-maze, novel object test, and the Morris water maze in mouse models of mild cognitive impairments. According to Du et al. [27], *A. sinensis* polysaccharide improved memory in AD rats via stimulating the BDNF/TrkB/CREB pathway. In a rat model of repeated cerebral ischemia, Nogami-Hara et al. [28] found that the Japanese *A. acutiloba* root (*Angelica* root) and yokukansan raised hippocampal acetylcholine levels, reduced apoptosis, and enhanced memory. Choi et al. [29] discovered that *A. tenuissima* Nakai reduced cognitive deficits in the Aβ1-42-infused mice by increasing BDNF expression and the phosphorylation of ERK1/2 and CREB, as well as inhibiting the neuronal loss and neuroinflammatory response. By inhibiting inflammation, apoptosis, and the NF-κB signaling pathway, Duan et al. [30] demonstrated that *A. sinensis* effectively rescued the symptoms of the Aβ-induced rat model of AD. APO also maintained the increase in spatial memory due to the key components significant to cognitive-enhancing effects (α-pinene, sabinene, *p*-cymene). The neuroprotective properties of α-pinene were revealed by Lee et al. [31], who improved Sco-induced memory impairment in C57BL/6 mice using Y-maze and passive avoidance tests. In addition, sabinene, one of the chemicals found in *Abies koreana* essential oil, was found to improve memory in mice suffering from Sco-induced amnesia [32]. Furthermore, p-cymene alleviated Aβ1-42-induced AD in a rat model via antioxidant and anti-inflammatory effects, as well as a direct antifibril impact [33]. Our findings establish a strong foundation for using the APO to treat memory loss and dementia.

### 2.4. Effects on Acetylcholinesterase Activity

To further understand the underlying mechanism of APO’s memory-enhancing activity in Sco-treated zebrafish, researchers looked at the levels of biochemical markers connected to cholinergic activities, such as acetylcholinesterase (AChE).

**Figure 4:**
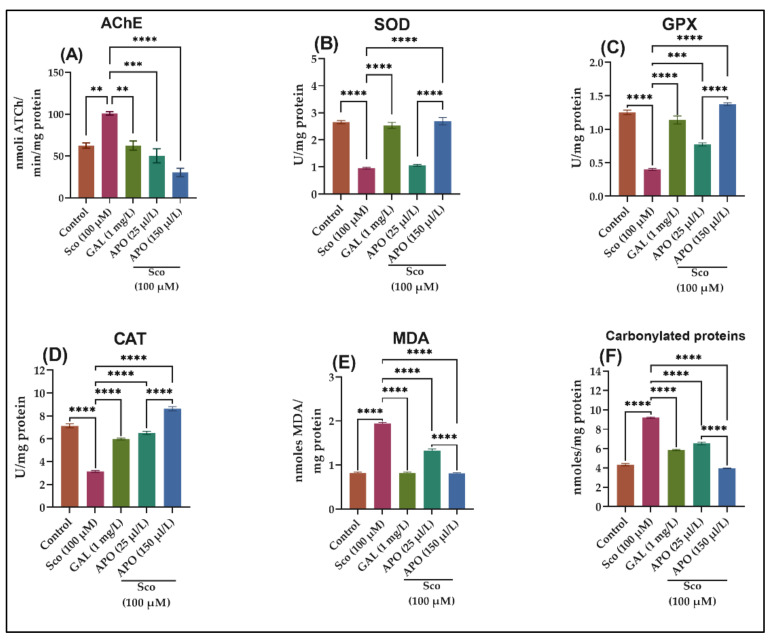
Effects of *Angelica purpurascens* essential oil (APO: 25 and 150 μL/L) on (**A**) AChE; (**B**) Superoxide dismutase (SOD); (**C**) Glutathione peroxidase (GPX) and (**D**) catalase (CAT)-specific activities; (**E**) malondialdehyde (MDA); (**F**) carbonylated protein levels. Data are represented by means ± S.E.M. (*n* = 10). ** *p* < 0.001, *** *p* < 0.0001, and **** *p* < 0.00001 (Tukey’s *post hoc* analyses). Galantamine (GAL, 1 mg/L) was used as reference positive drug.

A one-way ANOVA demonstrated that the treatment had a substantial overall effect on the AChE specific activity (F (4, 10) = 23.69, *p* < 0.0001)) (see Figure 4A). AChE activity was significantly decreased in the GAL group (*p* < 0.001) (Figure 4A) than that in the Sco group, showing that AChE markedly decreased, as similar to the control group. When compared to the control group, the Sco-induced zebrafish showed significantly higher AChE-specific activity (*p* < 0.001). In contrast, APO treatment significantly reduced AChE-specific activity dose-dependently (*p* < 0.0010 for 25 μL/L and *p* < 0.0010 for 150 μL/L) when compared to the Sco alone-treated groups.

Nowadays, acetylcholine levels are increased or protected by decreasing AChE, which is advantageous for treating AD [34]. The *A. purpurascens* has AChE inhibitory action according to supporting evidence. Due to the significant content of monoterpene hydrocarbons in the aerial parts, Karakaya et al. [10] reported that *A. purpurascens* (Avé-Lall.) Gilli. exhibited high AChE inhibitory activity and outstanding antioxidant potential. Ali et al. [35] found that dihydroxanthyletin-type coumarins from *A. decursiva* reduced AChE activity. Furthermore, Ali et al. [36] demonstrated that *A. decursiva* has antidiabetic and anti-AD properties by inhibiting AChE activity. Park et al. [37] have observed the memory-improving benefits of INM-176, a standardized ethanolic extract of *A. gigas* Nakai, in mice via AChE inhibition against Sco- or Aβ(1-42)-induced cognitive impairment. As a result, APO increased the nootropic impact in the Y-maze and NOR tests by reducing the cholinergic deficiencies produced by the Sco treatment.

### 2.5. Effects on Brain Oxidative Status

The one-way ANOVA revealed that the treatment had a significant impact on the brain oxidative status, for SOD (F (4,10) = 11.06, *p* < 0.0001) (Figure 4B), GPX (F (4, 10) = 13.67, *p* < 0.0001) (Figure 4C), CAT (F (4, 10) = 20.58, *p* < 0.0001) (Figure 4D), and elevated levels of lipid peroxidation (MDA) (F (4, 10) = 39.68, *p* < 0.0001) (Figure 4E) and protein oxidation (carbonylated proteins) (F (4, 10) = 47.28, *p* < 0.0001) (Figure 4F). As compared to Sco-treated animals, the GAL treatment significantly increased the brain SOD (*p* < 0.00001) (Figure 4B), GPX (*p* < 0.00001) (Figure 4C), and CAT (*p* < 0.00001) (Figure 4D)-specific activities and reduced the brain MDA (*p* < 0.00001) (Figure 4E) and carbonylated protein levels (*p* < 0.00001) (Figure 4F). In comparison to the control group, the Sco-exposed zebrafish showed a significant decrease of the specific activities of the antioxidant enzymes, such as SOD (*p* < 0.00001) (Figure 4B), GPX (*p* < 0.00001) (Figure 4C), and CAT (*p* < 0.00001) (Figure 4D) as well as elevated levels of MDA (*p* < 0.00001) (Figure 4E) and carbonylated proteins (*p* < 0.00001) (Figure 4F).

APO treatment increased antioxidant enzyme activity by decreasing the Sco-induced brain oxidative stress: SOD (*p* < 0.00001 for 150 /μLL) (Figure 4B), GPX (*p* < 0.0001 for 25 /μLL and *p* < 0.00001 for 150 /μLL) (Figure 4C), CAT (*p* < 0.00001 for 25 and 150 /μLL) (Figure 4D), and lowering measures of MDA (*p* < 0.00001 for 25 and 150 /μLL) (Figure 4E) and carbonylated proteins (*p* < 0.00001 for 25 and 150 /μLL) (Figure 4F) compared to Sco-treated zebrafish.

Oxidative stress exacerbates the cause of AD. In preclinical and clinical trials, increasing levels of oxidative stress during the latent period of the disease produces a rapid onset of cognitive impairment symptoms [38,39]. SOD and GSH levels in the antioxidant defense system are significantly reduced in mice with Sco-induced memory impairment and human patients with AD [40]. The protective properties of APO against oxidative stress produced by Sco in zebrafish were demonstrated in this study. Additionally, APO treatment increased antioxidant activity by raising the antioxidant enzymes SOD, GPX, and CAT, as well as lowering MDA and carbonylated protein levels in brain tissue. Our findings are backed up by research that shows *Angelica* reduces oxidative stress [41]. *A. sinensis* reduced vascular endothelial cell dysfunction by decreasing oxidative stress according to Yin et al. [42]. By decreasing myocardial fibrosis, lowering myocardial apoptosis, and alleviating oxidative stress, Song et al. [43] found that *A. sinensis* polysaccharide might prevent hypertensive heart disease. Furthermore, the significant amount of β-phellandrene (33.8%), sabinene (6.80%), α-pinene (5.30%), and α-phellandrene (4.20%) in APO could be linked to its antioxidant action. In an acute model of ifosfamide-induced hemorrhagic cystitis in mice, Gonçalves et al. [44] found that α-phellandrene reduced tissular damage, oxidative stress, and TNF-α levels. The therapeutic potential of α- and β-pinene against H_2_O_2_-stimulated oxidative stress was described by Salehi et al. [45]. According to the current findings, APO exhibits antioxidant properties and so could be a natural antioxidant alternative for therapeutic purposes.

### 2.6. Pearson Correlations between Behavioral and Biochemical Parameters

**Figure 5:**
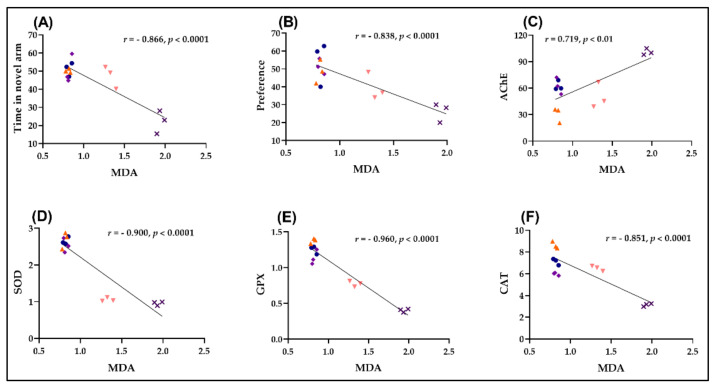
Correlation analyses between behavioral and biochemical parameters (Pearson’s correlation). Data expressed are time in the novel arm (s), preference (%), AChE (nmol/min/mg protein), SOD (U/mg protein), GPX (U/mg protein), CAT (U/mg protein), and MDA (nmol/mg protein). (**A**) Time in novel arm vs. MDA (*n* = 10, *r* = −0.866, *p* < 0.0001); (**B**) Preference (%) vs. MDA (*n* = 10, *r* = −0.838, *p* < 0.0001); (**C**) AChE vs. MDA (*n* = 10, *r* = 0.719, *p* < 0.01); (**D**) SOD vs. MDA (*n* = 10, *r* = −0.900, *p* < 0.0001); (**E**) GPX vs. MDA (*n* = 10, *r* = − 0.960, *p* < 0.0001); (**F**) CAT vs. MDA (*n* = 10, *r* = −0.851, *p* < 0.0001) in control (●), scopolamine (Sco) (**x**), galantamine (GAL) (♦), and APO ((▼) 25 and (▲) 150 /μLL) groups.

Pearson’s correlation coefficient (*r*) was used to explore the relationship between behavioral scores, enzymatic activities, and lipid peroxidation, which included time in the novel arm, preference, AChE, SOD, GPX, CAT, and MDA (Figure 5). With an *r* of −0.866 (Figure 5A) and –0.838 (Figure 5B), respectively, the time in the novel arm (Figure 5A) and the preference for the novel object (Figure 5B) showed a significant negative correlation to MDA, while SOD vs. MDA (Figure 5D), GPX vs. MDA (Figure 5E), and CAT vs. MDA (Figure 5F) showed a high negative correlation with an *r* of −0.900 (Figure 5D), −0.960 (Figure 5E), and −0.851 (Figure 5F). Furthermore, with an *r* of 0.719, a substantial positive connection between AChE vs. MDA was discovered (Figure 5C).

Karakaya et al. [10] discovered a link between natural APO chemicals and AChE inhibition. Türkuçar et al. [14] published a link between phenolic chemicals, essential oil composition, and APO antioxidant activity. Increased antioxidant enzyme activity and lower MDA (lipid peroxidation levels) are linked to better memory in Sco-treated zebrafish, indicating that APO has neuroprotective properties.

## 3. Materials and Methods

### 3.1. Plant Material and Essential Oil Preparation

Air-dried aerial parts of *Angelica purpurascens* (Avé-Lall.) Gilli. was collected in June 2019 from Tunceli, Turkey. The plant was authenticated by Prof. Dr. Eyup Bagci. Specimens (no. 2019HFU) were kept at the Herbarium of the Department of Biology, Firat University, Elazig, Turkey. The essential oil was extracted by hydrodistillation. The yield of the essential oil was 0.7% (*v*/*w*) and was stored at 4 °C until utilized.

### 3.2. Gas Chromatography–Mass Spectrometry (GC-MS/GC-FID) Analysis

The sample was analyzed by an Agilent 5973 N GC-MS system (Agilent Technologies, USA) with 6890 GC equipped with a flame ionization detector (FID) [46]. The GC conditions were as follows: injector temperature, 250°C; carrier gas (helium), flow rate 1 //mLmLmin. Oven temperature was initially 70 °C and then raised to 240 °C at a rate of 5 °C/min. The sample was diluted in n-hexane (1:100 *v*/*v*) and injected on a HP-5 MS column (30 m × 0.25 mm i.d., film thickness = 0.25 μm). The ionization energy was 70 eV and with a mass range of 35–425. The chemical compounds of the essential oil were identified by comparing their RI to those of n-alkanes (C8–C22) as external references, their retention times (RT), and their mass spectra with that reported in MS libraries (Wiley) [47].

### 3.3. Animals and Study Design

Sixty adult zebrafish (*Danio rerio*) (3–4 months old) of both sexes (50:50 male:female ratio) of the wild-type short-fin strain were obtained from an authorized commercial supplier (Pet Product S.R.L., Bucharest, Romania). The fish were kept with dechlorinated water (30 L tanks, 4 fish/L) at 26 ± 1 °C temperature, 7.0–7.2 pH, 7.2 mg O_2_/L, conductivity 1500–1600 μS cm^−1^ under 14-h light/dark cycle. They were fed twice daily with Norwin Norvitall flake (Norwin, Gadstrup, Denmark).

The experimental protocol was conducted according to the guidelines of the Directive 2010/63/EU of the European Parliament. Animal ethics no. 02/30.06.2020 was approved by the Ethics Committee on Animal Research of the Faculty of Biology, Alexandru Ioan Cuza University of Iași, Romania.

The zebrafish were divided into 6 groups (*n* = 10):

Group I: control

Group II: Sco (induced by scopolamine, 100 µM)

Group III: Sco + APO (25 µL/L) (induced by scopolamine and treated with APO 25 µL/L)

Group IV: Sco + APO (150 µL/L) (induced by scopolamine and treated with APO 150 µL/L)

Group V: Sco + IMP (20 mg/L) (induced by scopolamine and treated with imipramine, IMP, 20 mg/L)

Group VI: Sco + GAL (1 mg/L) (induced by scopolamine and treated with galantamine, GAL, 1 mg/L)

**Figure 6:**
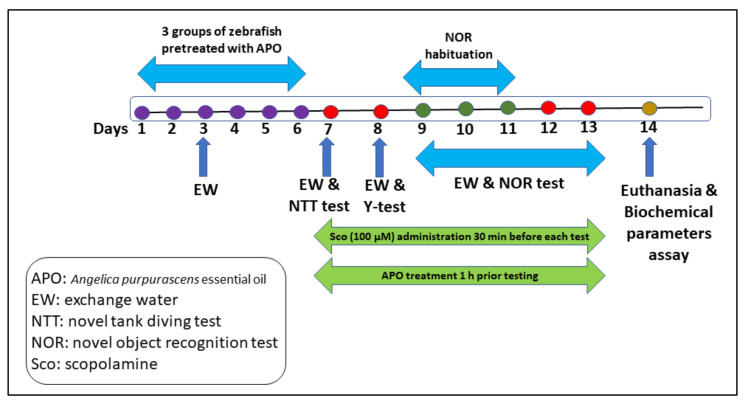
The experimental design of the study.

Treatment APO dosages [48] and Sco (100 µM) were chosen according to previous studies. APO (25 and 150 L/L) was diluted with 1% Tween-80 solution and given to zebrafish once daily for 13 days by immersion for 1 h, whereas Sco (100 µM) was given 30 min before each behavioral test. The control group was solely exposed to unchlorinated water containing a 1% Tween-80 solution. GAL and IMP were also given to the Sco-treated fish by immersion 30 min before the behavioral testing. The experimental design is outlined in Figure 6.

### 3.4. Behavioral Testing

In our research, zebrafish were individually recorded using an HD Webcam C922 camera (Logitech, Lausanne, Switzerland) and the videos were analyzed using ANY-maze^®^ software (Stoelting CO, Wood Dale, IL, USA).

#### 3.4.1. Novel Tank Diving Test (NTT)

The NTT is a specific test used for evaluating anxiety depression types of behavior in zebrafish, as described by Cachat et al. [49]. The NTT consisted of a 1.5 L trapezoidal tank (15.2 × 27.9 × 7.1 cm) divided by a virtual horizontal line into top and bottom sections. Zebrafish were individually assessed for 6 min, calculating the number of entries to the top, time spent in the top (s), average entry duration (s), total distance traveled in the top (m), distance traveled (m), freezing duration (s), and average velocity (m/s).

#### 3.4.2. Y-Maze Test

The response to novelty in zebrafish was evaluated using the Y-maze task [50]. The position in the Y-maze test was considered an index of memory [51]. Zebrafish were individually trained in a Y-maze glass aquarium (3 L) with three arms (25 × 8 × 15 cm) which were designated as the “start” arm (always open), the “novel” arm (open during the test trial), and the permanently open arm. During the first training session (5 min), the fish were individually placed in the start arm and the novel arm was closed. After 1 h, the second training session (5 min) began, and the fish were again placed in the start arm, but this time, the novel arm was opened. Time spent in novel arm (% of total arm time), total distance traveled (m), and turn angle (°) were the behavioral parameters evaluated in this test.

#### 3.4.3. Novel Object Recognition Test (NOR)

The NOR is a commonly used behavioral assay for investigating memory performance in zebrafish [52]. Briefly, zebrafish were subjected to 5 min of acclimation to the novel tank (30 × 30 × 30 cm filled with 6-cm water) for 3 consecutive days in the absence of the objects. On the fourth day, zebrafish were exposed for 10 min to two identical objects (training phase). One hour after the training phase, one of two identical objects (familiar objects, FO) was randomly replaced with a novel object (NO) and the interaction was monitored for 10 min (testing phase). The preference percentages were calculated as follows: [time of exploration of NO/time of exploration of FO + time of exploration of NO × 100].

### 3.5. Biochemical Study

After behavioral measures, the zebrafish were euthanized by immersion for 10 min in ice-cold water at 4 °C until the gill movements stopped [53]. Furthermore, the skull was removed, and the zebrafish brains were dissected out and gently homogenized with 0.1 M potassium phosphate buffer and 1.15% KCl in ice at pH 7.4 using a Potter Homogenizer (Cole-Parmer, Vermon Hills, IL, USA). Moreover, the homogenate sample was centrifuged for 15 min at 960 × *g* and the supernatant was used for estimation of the antioxidant enzyme activity and oxidative stress markers.

All procedures were carried out in conformity with the applicable rules and regulations. The acetylcholinesterase (AChE) activity was estimated in the brain supernatant according to the method of Ellman [54]. The superoxide dismutase (SOD, EC 1.15.1.1) activity was measured based on the Winterbourn protocol [55]. Catalase (CAT, EC 1.11.1.6) activity was analyzed by the Sinha method [56]. Glutathione peroxidase (GPX, E.C. 1.11.1.9) activity was estimated by the Sharma and Gupta method [57]. Carbonylated protein levels were assessed based on the protocol described by Oliver et al. [58] and modified through Luo and Wehr [59]. Lipid peroxidation (malondialdehyde level, MDA) was assessed by the method of Ohkawa [60]. The protein content in the brain supernatant was quantified through the method of Bradford [61].

### 3.6. Statistical Analysis

Data normality was analyzed by the Shapiro–Wilk test. All values are presented as mean ± standard error of means (SEM) (*n* = 10) and analyzed by one-way ANOVA and Tukey’s post hoc test was utilized for multiple comparisons. Statistical analysis was performed by GraphPad Prism 8.0 software (San Diego, CA, USA). Differences between groups were considered statistically significant at *p* < 0.05. Correlation between behavioral results, enzymatic activities, and lipid peroxidation was estimated by the Pearson correlation coefficient (*r*).

## 4. Conclusions

In behavioral testing, APO reduced Sco-induced anxiety and cognitive deficits. The mechanism was to increase ACh by suppressing AChE activity while also reducing oxidative stress, resulting in better cognition and anxiolytic activity. For the first time in this investigation, APO was proven as a promising anti-AD candidate, providing fresh information for its future usage in the treatment of AD-related conditions.

## Figures and Tables

**Table 1:** Compositional analysis of *Angelica purpurascens* essential oil.

Compounds ^a^	Lit. RI ^b^	Exp. RI ^c^	Area (%) ^d^
α-Pinene	939	935	5.3 (±0.00)
Sabinene	976	975	6.8 (±0.01)
δ-3-Carene	997	995	1.4 (±0.00)
α-Phellandrene	1002	1005	4.2 (±0.02)
*p*-Cymene	1020	1027	3.8 (±0.00)
Limonene	1024	1030	1.6 (±0.00)
β-Phellandrene	1025	1023	33.8 (±0.01)
α-Terpineol	1186	1190	1.1 (±0.00)
Dodecanal	1389	1380	1.4 (±0.00)
β-Elemene	1391	1389	0.5 (±0.00)
Aromadendrene	1449	1445	1.9 (±0.00)
Germacrene-D	1480	1478	4.5 (±0.00)
Total identified			66.30

^a^ Compounds are listed in their order of elution from HP-5 MS column; ^b^ Literature retention index (Lit. RI); ^c^ Retention index (Exp. RI) relative to standard mixture of *n*-alkanes on HP-5 MS column; ^d^ Relative peak area percentage (averages of three determinations).

## Data Availability

The data presented in this study are available on request from the corresponding author.
